# The Association of Body Mass Index and Fat Mass with Health-Related Physical Fitness among Chinese Schoolchildren: A Study Using a Predictive Model

**DOI:** 10.3390/ijerph20010355

**Published:** 2022-12-26

**Authors:** Qiang Wang, Hongzhi Guo, Sitong Chen, Jiameng Ma, Hyunshik Kim

**Affiliations:** 1 College of Sports Science, Shenyang Normal University, Shenyang 110034, China; 2 Graduate School of Human Sciences, Waseda University, Tokorozawa 359-1192, Japan; 3 Institute for Health and Sport, Victoria University, Melbourne, VIC 3011, Australia; 4 Faculty of Sports Science, Sendai University, Shibata 989-1693, Japan

**Keywords:** speed, flexibility, coordination, muscular endurance, endurance, schoolchildren, Chinese

## Abstract

Body fat mass (FM) has advantages over body mass index (BMI) in terms of accuracy of fitness assessment and health monitoring. However, the relationship between FM and fitness in Chinese children has not yet been well studied. This study aimed to investigate the relationship between health-related physical fitness, BMI, and FM, which was estimated using a predictive model among elementary schoolchildren in China. This cross-sectional study included 2677 participants (boys, 53.6%; girls, 46.4%) who underwent anthropometric measurements (height, weight, BMI, and FM) and five health-related fitness tests: 50-m sprint (speed), sit and reach (flexibility), timed rope-skipping (coordination), timed sit-ups (muscular endurance), and 50-m × 8 shuttle run (endurance). In boys, BMI showed a positive correlation with speed (*p <* 0.001) and endurance (*p <* 0.006) tests and a negative correlation with flexibility (*p <* 0.004) and coordination (*p <* 0.001) tests. In girls, a positive correlation between speed (*p <* 0.001) and endurance (*p <* 0.036) tests was observed. Both BMI and FM (estimated using the predictive model) were strongly associated with the health-related physical fitness of elementary schoolchildren. Our findings indicate that health-related physical fitness was similarly affected by FM and BMI. As FM can be quantified, it could therefore be used to develop strategies and intervention programs for the prevention and management of obesity in children.

## 1. Introduction

Obesity is a chronic progressive disease [1] and a serious public health concern worldwide [2]. The global prevalence of obesity has increased twofold since 1980 [3]. Annually, 2.8 million people die from being overweight or obese, as well as due to the complications of obesity [4]. China is not an exception; the prevalence of overweight and obesity in Chinese children has rapidly increased over the past several decades [5]. Overweight and obesity are characterized by an excessive accumulation of fatty tissues, which may affect an individual’s ability to exercise [6] as well as brain health [7] and may increase the risk of developing chronic diseases, such as type 2 diabetes [8]. Therefore, the implementation of effective preventive strategies against overweight and obesity in children is an essential public health agenda.

Fitness is a comprehensive measure of an individual’s ability to perform physical activity and exercise and is known as an important health indicator [9]. Generally, health-related physical fitness encompasses cardiorespiratory endurance, muscle strength and endurance, flexibility, and body composition [10]. Overweight and obese children are likely to have poorer health-related physical fitness, including physical exercise performance and cardiorespiratory endurance [11]. Additionally, based on a study suggesting that increased muscle strength and endurance were potentially linked to a lower risk of obesity [12], health-related physical fitness may be a reliable predictor of the risk of illness and may aid in preventing overweight and obesity in the youth [13].

Body mass index (BMI) is a widely used measure for weight status classification and is reliable for obesity assessment in the youth [14]. Being overweight and obese, as indicated by the BMI, negatively influences the fitness level of children [15,16,17]. Moreover, BMI can be easily used when more accurate techniques, such as dual-energy X-ray absorptiometry (DEXA), are unavailable. However, BMI also has several limitations [18]. In particular, BMI is an estimate based on height and weight [19] and does not differentiate between muscle mass and fat mass (FM); thus, it cannot be used to determine FM [20]. Compared with BMI, FM is more strongly associated with the risk of long-term type 2 diabetes in children [21] and negatively affects health-related physical fitness [22]. Recently, a greater emphasis has been placed on body fat, the accuracy of which is superior to that of BMI in fitness assessment and health monitoring [23]. 

A recent study suggested a “predictive model for fat mass” to estimate body FM in children based on height, weight, sex, age, and ethnicity [24]. The model is based on simple anthropometric measurements without the need for complex body composition assessment. Further, FM assessment has been proposed to be as accurate as DEXA and bioelectrical impedance analysis [25]. Although the relationship between FM and fitness has been the subject of some studies [22,25], this relationship remains to be elucidated in Chinese children. The prevalence of obesity and poor physical fitness among Chinese children continues to increase significantly [26,27,28], and investigating the impact of obesity on health-related physical fitness is important if China were to achieve its target of 25% of its school-aged children meeting the criterion of “excellent” fitness level by 2030 [29]. Therefore, the present study aimed to investigate the relationship between health-related physical fitness, BMI, and FM (estimated using the predictive model) and provide direction for future research on enhancing physical fitness and reducing obesity in elementary school-aged students in China.

## 2. Materials and Methods

### 2.1. Study Design and Participants

This cross-sectional study analyzed part of the data collected from a convenient sampling of elementary schools in northeastern China that had consented to participate in the “Study for the Improvement of Life Habits in East Asian Children” project [30,31]. The inclusion criteria were as follows: elementary school-aged students aged 7–12 years without physical or mental disorders. Prior to participation, the children and their guardians were provided with a written document describing the study and a written consent form. Data of the children who consented to participate were analyzed. A total of 2732 elementary school-aged students residing in Liaoning Province (northeast China) participated and were assessed for fitness in October 2021 by school grade and sex. Children who did not consent to study participation (*n =* 18), did not undergo all fitness tests (*n =* 13), and had errors in their data (such as missing information in the online survey, *n =* 24) were excluded from the analysis. Finally, the data of 2677 children (boys, 53.6%; girls, 46.4%) were analyzed. The study received prior approval from the Ethics Committee of the Faculty of Sports Science, Sendai University (IRB number: SU2201-05).

### 2.2. Anthropometric Measurements

Anthropometric measurements were performed by teachers, school nurses, and researchers. Prior to height and weight measurements, the children were instructed to take their shoes off. Height (cm) was measured to the nearest 0.1 cm, whereas weight (kg) was examined to the nearest 0.1 kg using a portable instrument (GMCS-IV; Jianmin, Beijing, China) [32]. Based on the height and weight results, the BMI was calculated as weight in kilograms divided by height in meters squared (kg/m^2^). To compare the weight among different age groups, BMI percentiles were categorized based on sex and age according to the 2000 Centers for Disease Control and Prevention (CDC) growth charts for the United States using the following categories: underweight, healthy weight, overweight, and obese [33]. The BMI z-score was calculated based on sex and age in months according to the 2000 CDC growth charts for the United States [33]. Subsequently, FM was calculated using the following formulae [24]:

FM = height − exp(0.3073 × height^2^ − 10.0155 × weight^−1^ + 0.004571 × weight − 0.9180 × ln(age) + 0.6488 × age^0.5^ + 0.04723 × male + 2.8055) (1)

FM = height − exp(0.3073 × height^2^ − 10.0155 × weight^−1^ + 0.004571 × weight − 0.9180 × ln(age) + 0.6488 × age^0.5^ + 0.04723 × female + 2.8055) (2)

(exp = exponential function, ln = natural logarithmic transformation, male = 1, female = 0).

Microsoft Excel formulae were used to rapidly compute FM and can be applied in clinical practice and the public health field.

### 2.3. Health-Related Physical Fitness

To assess the health-related physical fitness, the test items in the “2014 National Student Physical Fitness Standard in China” recommended by the Chinese Ministry of Education were selected and classified into four categories according to sex [34]. The test-retest reliability across all assessments employed in the current study was ICC > 0.90, which was determined acceptable [35].

The health-related physical fitness was measured as follows:

50-m sprint (speed): In this test, the time from the start signal to the moment at which the torso (not head, shoulders, hands, or legs) reached the goal line was measured. Participants performed the test once (a single maximum sprint), and running times were recorded to the nearest 0.1 s. Participants of all ages performed this test.

Sit and reach (flexibility): This test was used to assess flexibility. The participants were told to sit and stretch their hands forward. They were instructed to sit with their feet placed against a vertical support and to reach their hands forward as far as possible along the measuring line while paying attention not to bend the knees. The test was performed twice, and the farther distance (measured to 0.1 cm) of the two was recorded. Participants of all ages performed the test.

Timed rope-skipping (coordination): A rope of appropriate size and length was used. The participants were asked to skip the rope continuously for 1 min, and the total number of jumps was recorded. The children were instructed to always jump with two feet, and a jump was counted only if both feet touched the ground. Participants of all ages performed the test.

Timed sit-ups (muscular endurance): For this test, the participants held the starting posture of knees bent, feet flat, and hands behind the head with fingers interlaced. Subsequently, they were instructed to lift the torso until the elbows touched the thighs and then return to the starting position so that the shoulders touched the mat. They performed sit-ups for 1 min, and the number of sit-ups performed in the accurate posture was recorded. Only participants from the 3rd to 6th grades performed the test.

50-m × 8 shuttle run (endurance): This test required the children to run back and forth 8 times along a straight track line between two poles set 50 m apart. They were instructed to run at their maximum speed and, at the end of the track line, turn around at a pole in a counterclockwise direction and run back to the starting line. Each child performed a single trial, and his or her time was recorded to the nearest second. This measure was assessed only in primary schoolchildren (grades 5 and 6).

### 2.4. Demographic Variables

A questionnaire was used to survey the demographic variables of the participants, such as sex, age, height, weight, grade in school, and ethnicity [36,37].

### 2.5. Statistical Analysis

Data from 2677 Chinese schoolchildren (1434 boys and 1243 girls) who provided complete information on the study variables were analyzed using four models. 

In the first model, the difference in health-related physical fitness variables was analyzed by age, height, weight, BMI, and FM. The independent *t*-test and chi-square test were conducted for continuous and categorical variables, respectively. 

In the second model, a one-way analysis of variance was performed by classifying the body type of Chinese schoolchildren into one of four categories by sex to compare the mean values of health-related physical fitness variables. Subsequently, Tukey’s post hoc test was used to examine the statistical significance of between-group differences. 

In the third model, the relationship between BMI and FM variables was investigated by applying the forced input method using univariate linear regression analysis for health-related fitness variables according to sex.

In the fourth model, logistic regression analysis was conducted on the four categories of each health-related physical fitness variable to examine their association with BMI z-score and FM by sex. The four health-related physical fitness categories were determined as follows: scores were first computed by applying weights corresponding to different school grades and sexes, which were defined in the 2014 revision of the Chinese National Student Physical Fitness Standard. Subsequently, health-related physical fitness scores were grouped into Q1 (90.0 points or higher), Q2 (80.0–89.9), Q3 (60.0–79.9), and Q4 (<60) [34]. Additionally, age, height, weight, and ethnicity were used as covariates. The Q1 group for each measure was the reference group for this analysis. Odds ratios (ORs) and 95% confidence intervals (CIs) were calculated for each variable. Inferential statistics were performed using IBM SPSS 26.0 (IBM, Armonk, NY, USA), and the level of significance was set at *p <* 0.05.

## 3. Results

Table 1 presents the participants’ height, weight, BMI, and health-related physical fitness performance according to sex. The BMI and BMI z-score were higher in boys than in girls (BMI: 18.1 ± 3.6 vs. 17.6 ± 3.4 in boys and girls, respectively [*p <* 0.001]; BMI z-score: 0.4 ± 1.2 vs. 0.1 ± 1.2 in boys and girls, respectively [*p <* 0.001]). The rates of overweight and obesity were higher in boys than in girls (overweight: 19.8% vs. 16.4% in boys and girls, respectively [*p <* 0.001]; obesity: 16.0% vs. 7.9% in boys and girls, respectively [*p <* 0.001]). Additionally, compared with girls, boys were faster in the 50-m sprint (10.2 s vs. 10.6 s, *p <* 0.001) and performed more sit-ups in the timed sit-up test (31.8 times vs. 26.3 times, *p <* 0.001). However, girls scored higher than boys in the sit and reach test (8.8 cm vs. 11.7 cm, *p <* 0.001) and timed rope-skipping (90.7 jumps vs. 95.7 jumps, *p <* 0.001).

Table 2 shows the participants’ mean health-related physical fitness levels based on BMI categories. In both sexes, FM steadily increased from underweight to obese individuals. For each body type, FM was higher in boys than in girls (*p <* 0.001). Among boys, the healthy weight group showed a significantly better performance than the over-weight and obese groups in the 50-m sprint, sit and reach, timed rope-skipping, and 50-m × 8 shuttle run tests. Among girls, the healthy weight group performed significantly better in the 50-m sprint, timed rope-skipping, and 50-m × 8 shuttle run (Table 2).

Table 3 shows the relationship between BMI and health-related physical fitness adjusted for covariates such as sex, age, and ethnicity. In boys, a positive correlation in the 50-m sprint (*p <* 0.001) and 50-m × 8 shuttle run (*p <* 0.006) and a negative correlation in the sit and reach (*p <* 0.004) and timed rope-skipping (*p <* 0.001) tests were observed. In girls, a positive correlation was observed in the 50-m sprint (*p <* 0.001) and 50-m × 8 shuttle run (*p <* 0.031) tests. In addition, the analysis conducted to examine the relationship between FM and health-related physical fitness using the predictive model for FM showed that the same health-related physical fitness tests significantly correlated with BMI in both boys and girls (Table 3).

**Figure 1:**
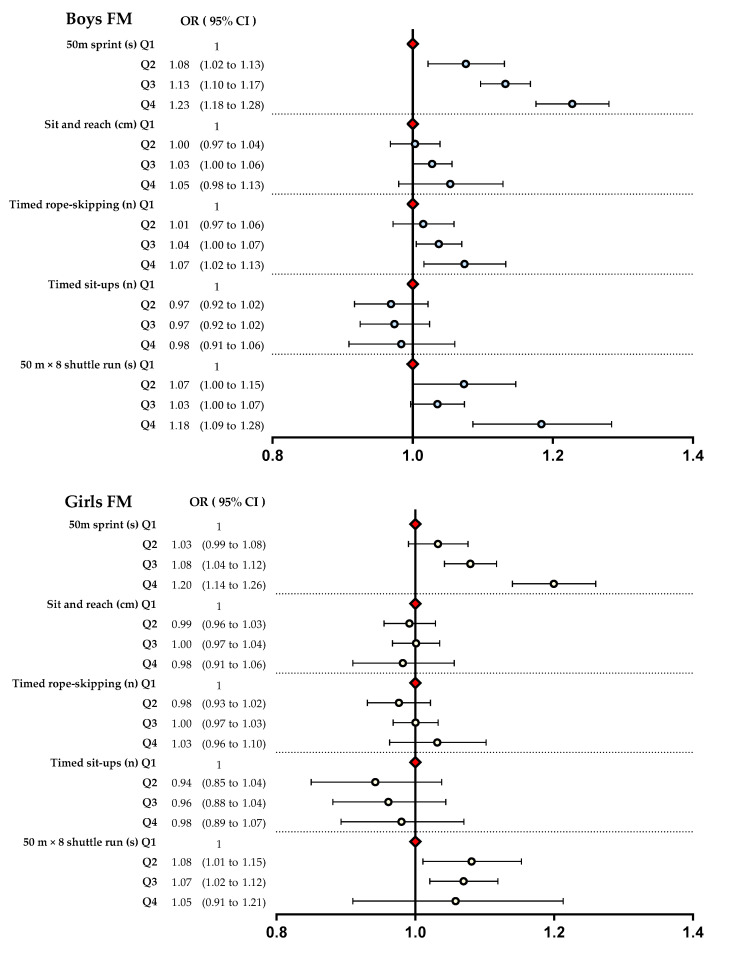
Logistic regression of the association between FM and health-related physical fitness in Chinese schoolchildren. Q1: 90.0 points or higher, Q2: 80.0–89.9, Q3: 60.0–79.9, Q4: <60. FM, fat mass.

Figure 1 illustrates the multivariate analysis of the adjusted ORs of FM according to sex for the four health-related physical fitness categories. In the 50-m sprint, the risk of obesity was the highest in Q4, with Q1 as the reference, in both boys and girls (boys, Q4: OR, 1.23, 95% CI, 1.18–1.28; girls, Q4: OR, 1.20, 95% CI, 1.14–1.26). Regarding timed rope-skipping, the risk of obesity was the highest in Q4 compared with Q1 in boys alone (Q4: OR, 1.07, 95% CI, 1.02–1.13). In the 50-m × 8 shuttle run, the risk of obesity in boys was the greatest in Q4 (OR, 1.18; 95% CI, 1.09–1.28); in girls, the risk was significantly greater in Q3 (OR, 1.07; 95% CI, 1.02–1.12).

## 4. Discussion

This is the first study conducted in an Asian country to investigate the relationship among BMI, FM (estimated using the predictive model), and health-related physical fitness in elementary schoolchildren in China. Our findings are relevant to researchers and policy makers developing effective preventive strategies against overweight and obesity as well as programs for increasing health-related physical fitness in elementary schoolchildren.

Our results support previous studies [10,16,38] and suggest that being overweight and obese may, prospectively, decrease health-related physical fitness. Likewise, a link between changes in health-related physical fitness and overweight/obesity was confirmed in a 4-year longitudinal study, which reported that the health-related physical fitness level decreased in children who gained weight and that the risk of overweight or obesity was higher in children with a low fitness level compared with those with a high fitness level at baseline [39]. Additionally, in the current study, performance in the 50-m sprint, timed rope-skipping, and 50-m × 8 shuttle run decreased with increasing FM. Similarly, the relationship between BM and health-related physical fitness was significant even according to the multivariate analysis in which the effects were adjusted for covariates. Similar to a previous study [40], in the analysis of the FM and BMI categories, the risk was higher in girls than in boys. In the 50-m sprint and 50-m × 8 shuttle run, FM was the highest in Q4 (the lowest health-related physical fitness level) in both boys and girls. Our findings suggest that the low explosive power in the lower limbs is associated with an increased risk of overweight and obesity in boys and girls. The 50-m sprint, which is a high-intensity physical activity, is a test to assess the explosive power of leg muscles. Because most FM is stored in the buttocks and legs, the skeletal muscles are overloaded and physical activity is reduced, causing movement dysfunction and limitations in performing daily activities [41]. With respect to the link between FM (estimated using the predictive model) and cardiorespiratory endurance, a previous study reported that muscular strength in the lower body and cardiorespiratory endurance were negatively associated with total and central body fat in children [42], and our finding is supported by previous studies [43,44]. Aerobic exercise is effective in improving cardiorespiratory endurance [45]; thus, increasing cardiorespiratory endurance would be most beneficial and effective in preventing and managing obesity in children. Additionally, muscle mass and body FM are linked to carbohydrate metabolism [46]. Increased body FM elevates the blood glucose level on an empty stomach and lowers the homeostatic model assessment for insulin resistance (HOMA-IR), triglyceride, and high-density lipoprotein cholesterol levels, whereas increased fat-free mass affects the HOMA-IR, triglyceride, and low-density lipoprotein cholesterol levels; furthermore, muscle strength and endurance exercises influence blood lipid levels and insulin sensitivity, suggesting that an uncontrolled blood glucose level may reduce the endurance level [47,48]. Thus, our findings suggest that overweight and obesity affect activities of daily living in children. Accordingly, for children to achieve improvements in health-related physical fitness, public health efforts to develop and implement policies for school physical education are necessary.

In this study, a correlation between timed rope-skipping and FM was noted only among boys. Timed rope-skipping is a whole-body exercise requiring rhythm, coordination, agility, speed, and power [49]. Particularly, it is a high-intensity aerobic exercise requiring over 70% of the maximal oxygen consumption. To walk or jog, a large field is required due to the need for a safe course and paved path, but rope-skipping can be performed even in a small area. Thus, it is suggested that physical activity (PA) intervention using rope-skipping be provided in and outside of the school environment to increase children’s PA participation and improve their overall physical strength [50]. Additionally, boys generally performed better than girls in most of the health-related physical fitness tests. The performance of girls in the sit and reach and timed rope-skipping tests was not significantly associated with FM. Because girls generally have less fat in the waist region than boys, they have more flexibility for stretching [51]. Additionally, it is necessary for girls to engage in a variety of sports as they tend to choose non-contact sports, such as rope-skipping and acrobatic exercises [52].

The performance in the sit and reach and timed sit-ups tests was associated with neither BMI nor FM (Figure S1). The findings of previous studies are conflicting. In studies conducted on Asian children, both positive [16] and negative [38] correlations between flexibility and obesity were observed. In Western children, flexibility and obesity were positively correlated in most studies [53,54,55]. A plausible explanation for this is that, unlike endurance, agility, and explosive power, movements involving flexibility are associated with body shape, which is influenced by weight status [56]. In the future, it would be worthwhile to further explore fundamental physiological mechanisms. Physical activity in daily lives is an important factor in preventing an increase in FM in children [57]. According to a previous study, the greater the physical activity during childhood, the lower the FM up to 11 years of age [58].

When interpreting our findings, the following limitations of the study should be considered. First, as this was a cross-sectional study, a causal relationship among BMI, FM, and health-related physical fitness could not be determined. A longitudinal or intervention study should be conducted to clarify such a relationship. Second, our study was conducted on students from northeast China, and our sample may not be representative of the entire population of elementary school-aged students in China. In addition, the survey area is located in a northern city with the fourth highest childhood obesity rate in the country, bringing into question the generalizability of results. Third, confounding factors for obesity such as genetics, lifestyle, and dietary habits were not investigated. Nevertheless, our findings are in line with those of previous studies and demonstrate the practical applicability of the novel formula (predictive model for FM) in estimating FM based on a physical fitness testing table for children in China. In the future, the scope of research should be expanded to using instruments such as DEXA and testing the accuracy of these modalities.

## 5. Conclusions

The current study showed that BMI and FM estimated using the formula for the predictive model for FM were strongly associated with health-related physical fitness in elementary schoolchildren. Regarding the relationship between the four health-related physical fitness categories and BMI, the performance in the 50-m sprint, timed rope-skipping, and 50-m × 8 shuttle run correlated with BMI in boys. Furthermore, the performance in the 50-m sprint and 50-m × 8 shuttle run significantly correlated with BMI in girls. Our findings indicate that health-related physical fitness was similarly affected by FM and BMI. Moreover, because FM can be quantified, it could serve as the basis for strategies addressing decreased health-related physical fitness and for the development of effective intervention programs in the future.

## Figures and Tables

**Table 1:** Participant characteristics.

Variables	Total (*n* = 2677)	Boys (*n* = 1434)	Girls (*n* = 1243)	*p*-Value
Age (*n*, %)				
7	497 (18.6%)	267 (18.6%)	230 (18.5%)	0.745
8	508 (19.0%)	270 (18.8%)	238 (19.1%)	
9	504 (18.8%)	274 (19.1%)	230 (18.5%)	
10	444 (16.6%)	225 (15.7%)	219 (17.6%)	
11	394 (14.7%)	212 (14.8%)	182 (14.6%)	
12	330 (12.3%)	186 (13.0%)	144 (11.6%)	
Height (cm: mean, SD)	138.7 ± 11.7	139 ± 11.9	138.2 ± 11.6	0.061
Weight (kg: mean, SD)	35.0 ± 10.5	36.2 ± 10.8	34.5 ± 10.0	<0.001
Race and ethnicity (Han: mean, %)	2116 (79.0%)	1143 (79.7%)	973 (78.3%)	0.365
BMI (kg/m^2^: mean, SD)	17.9 ± 3.5	18.1 ± 3.6	17.6 ± 3.4	<0.001
BMI z-score	0.3 ± 1.2	0.4 ± 1.2	0.1 ± 1.2	<0.001
BMI percentile ^a^				
Underweight	175 (6.7%)	83 (6.0%)	92 (7.5%)	<0.001
Normal	1639 (62.8%)	806 (58.2%)	833 (68.2%)	
Overweight	475 (18.2%)	275 (19.8%)	200 (16.4%)	
Obesity	319 (12.2%)	222 (16.0%)	97 (7.9%)	
FM (kg) ^b^	10.1 ± 5.2	10.0 ± 5.3	10.4 ± 5.1	0.052
Health-related physical fitness				
50-m sprint (s)	10.4 ± 1.5	10.2 ± 1.4	10.6 ± 1.6	<0.001
Sit and reach (cm)	10.1 ± 5.5	8.8 ± 5.3	11.7 ± 5.3	<0.001
Timed rope-skipping (*n*)	93.0 ± 35.8	90.7 ± 35.4	95.7 ± 36.1	<0.001
Timed sit-ups (*n*)	29.2 ± 9.1	31.8 ± 8.3	26.3 ± 9.1	<0.001
50-m × 8 shuttle run (s)	110.6 ± 16.9	109.5 ± 16.9	111.8 ± 16.7	0.065

Abbreviations: SD, standard deviation; FM, fat mass. ^a^ BMI percentiles of the participants were classified according to the age criteria of the Centers for Disease Control and Prevention growth charts for the United States [33]. ^b^ FM was calculated using the height-weight equation [24]. *p*-values were calculated using the t-test for continuous variables and the chi-square test for categorical variables.

**Table 2:** Health-related physical fitness measurements according to BMI categories.

Variables	Underweight	Normal	Overweight	Obesity	*p*-Value	Tukey’s Post Hoc Test
Boys						
FM (kg)	4.2 ± 0.6	7.7 ± 2.6	13.3 ± 3.6	17.8 ± 4.9	<0.001	a < b < c < d
50-m sprint (s)	10.2 ± 1.3	9.9 ± 1.4	10.4 ± 1.3	10.9 ± 1.3	<0.001	b < c; a, b, c < d
Sit and reach (cm)	8.9 ± 5.7	9.1 ± 5.2	8.7 ± 5.6	7.7 ± 5.3	0.004	b < d
Timed rope-skipping (*n*)	90.7 ± 33.1	94 ± 35.1	87.5 ± 35.4	82.5 ± 34.8	<0.001	b < d
Timed sit-ups (*n*)	30.5 ± 8.0	32.4 ± 8.0	31.6 ± 9.0	31.3 ± 8.5	0.262	n.s
50-m × 8 shuttle run (s)	109.9 ± 17.2	107.2 ± 16.8	112.4 ± 16.6	114.4 ± 16.3	0.011	b < d
Girls						
FM (kg)	4.9 ± 0.8	8.9 ± 3.1	15.4 ± 3.9	19.3 ± 5.0	<0.001	a < b < c < d
50-m sprint (s)	10.7 ± 1.5	10.4 ± 1.6	10.9 ± 1.5	11.5 ± 1.4	<0.001	b < c; a, b, c < d
Sit and reach (cm)	11.5 ± 5.9	11.8 ± 5.2	12.2 ± 5.2	11.1 ± 5.5	0.337	n.s
Timed rope-skipping (*n*)	96.3 ± 37.9	97.5 ± 35.1	92.4 ± 37.7	88.1 ± 36.6	0.043	n.s
Timed sit-ups (*n*)	24.2 ± 8.4	26.9 ± 9.1	26.1 ± 9.1	25.5 ± 9.4	0.100	n.s
50-m × 8 shuttle run (s)	112.3 ± 15.4	110.2 ± 16.8	116.7 ± 15.9	118.1 ± 16.4	0.045	n.s

Abbreviations: BMI, body mass index; FM, fat mass; n.s, not significant. BMI values are expressed as mean ± standard deviation. BMI percentiles were classified according to the age criteria of the Centers for Disease Control and Prevention growth charts for the United States [34].

**Table 3:** Results of the linear regression analyses of BMI, FM, and health-related physical fitness.

Variables	BMI ^a^			FM ^b^		
	B	95% CI	*p*	B	95% CI	*p*
Boys						
50-m sprint (s)	0.17	(0.14, 0.24)	<0.001	0.21	(0.04, 0.07)	<0.001
Sit and reach (cm)	−0.08	(−0.57, −0.11)	0.004	−0.10	(−0.16, −0.04)	<0.001
Timed rope-skipping (*n*)	−0.08	(−3.66, −0.92)	<0.001	−0.11	(−1.06, −0.37)	<0.001
Timed sit-ups (*n*)	0.01	(−0.42, 0.50)	0.865	−0.03	(−0.15, 0.06)	0.370
50-m × 8 shuttle run (s)	0.14	(0.57, 3.47)	0.006	0.16	(0.16, 0.72)	0.002
Girls						
50-m sprint (s)	0.07	(0.04, 0.15)	<0.001	0.11	(0.02, 0.05)	<0.001
Sit and reach (cm)	0.00	(−0.25, 0.26)	0.955	−0.01	(−0.07, 0.05)	0.770
Timed rope-skipping (*n*)	−0.02	(−2.10, 0.90)	0.433	−0.01	(−0.48, 0.28)	0.596
Timed sit-ups (*n*)	0.03	(−0.32, 0.75)	0.433	−0.02	(−0.15, 0.10)	0.675
50-m × 8 shuttle run (s)	0.12	(0.17, 3.42)	0.031	0.13	(0.04, 0.71)	0.027

Abbreviations: BMI, body mass index; FM, fat mass; CI, confidence interval. Standardized regression coefficients were adjusted for age, sex, race, and ethnicity. ^a^ BMI percentiles were classified according to the age criteria of the Centers for Disease Control and Prevention growth charts for the United States [33]. ^b^ FM was calculated using the height-weight equation [24].

## Data Availability

Data are contained within the article.

## Supplementary Materials

The following supporting information can be downloaded at: https://www.mdpi.com/article/10.3390/ijerph20010355/s1, Figure S1. Logistic regression of the association between BMI and health-related physical fitness in Chinese schoolchildren.
