# Supplementary material for: The Association of Body Mass Index and Fat Mass with Health-Related Physical Fitness among Chinese Schoolchildren: A Study Using a Predictive Model
Source: Int J Environ Res Public Health. 2022 Dec 26;20(1):355. doi: 10.3390/ijerph20010355 (PMC9819089; doi:10.3390/ijerph20010355)
Supplement: Supplementary file 1 [file ijerph-20-00355-s001.zip › ijerph-2074257-supplementary.pdf]

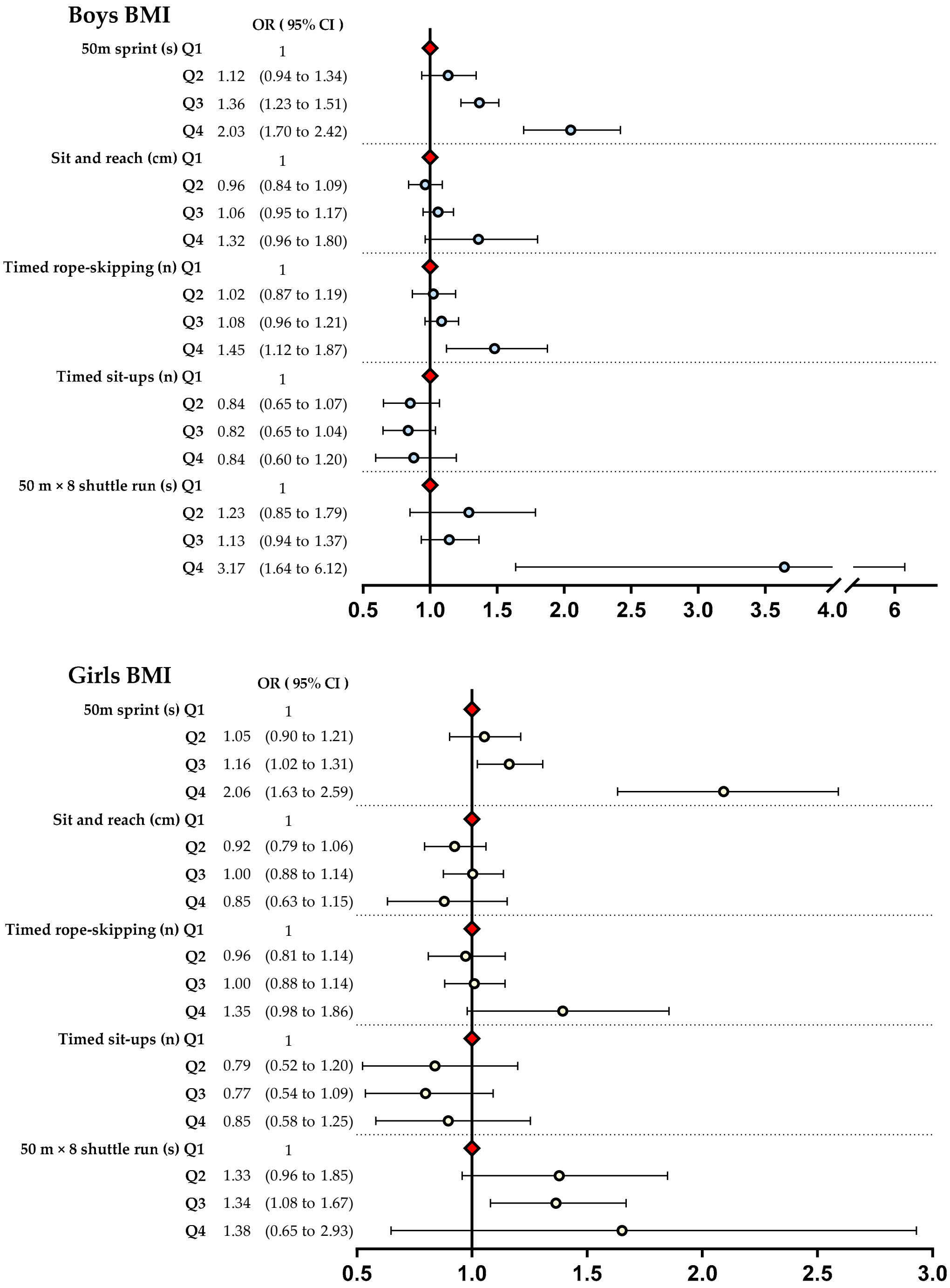

Figure S1. Logistic regression of the association between BMI and health-related physical fitness in Chinese schoolchildren. Q1: 90.0 points or higher, Q2: 80.0–89.9, Q3: 60.0–79.9, Q4: <60. FM, fat mass.
